# The Southern Dental Association Meeting at New Orleans

**Published:** 1870-05

**Authors:** 


### EDITORIAL DEPARTMENT.

*The Southern Dental Association Meeting at New Orleans* we learn, was one of the most interesting ever held in this country. We expect to publish a full report of the proceedings in the June No. of the Journal. As the meeting was held on the 13th 14th 15th and 16th of last month, it was impossible for us to obtain the proceedings in time for publication in the present number. That the members of the profession in attendance were well entertained by their brethern of New Orleans is evident from the following account of a dinner given them at the Washington Hotel, Lake End:

"The party with their invited guests, among whom were a number of ladies. left the city on the 2 o'clock Ponchartrain cars, and sat down at table at 4½ o'clock. The dinner considering the short notice given for its preparation, was nicely served, the guests from Northern latitudes seeming to be particularly charmed with the profusion of roses and other floral decorations. Among the profession present from the far interior, there were some who had never tasted an olive, a shrimp, a crayfish or an artichoke, but who, nevertheless, indulged in each and in all to a greater or less extent, acquiring in some instances a wonderful penchant for the, to them, novel edibles. Strawberries, bananas and other native and tropical fruits were specially enjoyed by the assembled company.

In the course of the entertainment toasts were offered and happily replied to by Dr. Frederick Knapp, Dr. Taft, of Cincinnati, Dr. Casadavant, Dr. Thurber and Dr. Walker, of the profession and by members of the press present.

Mr. Hape, of Atlanta, of Dental Depot fame, was presented with a splendid gold headed cane, by the Southern Dental Association, Dr. Morgan, of Nashville, making the presentation.

Both the presentation address and the reply were exceedingly felicitous. When the writer left the party, at 8 o'clock, they were still enjoying themselves, the ladies sipping their ices, while the gentlemen replenished their glasses between toasts with sparkling champagne. Before dinner was announced, the ladies of the party, with their escorts, went out for a stroll through the gardens of the hotel, and were much delighted with the show of flowers and the glimpses which they got of the lake and the vessels upon it, whose white sails were bellying out with the fresh southeaster."

The Reporter of the New Orleans Picayune writes as follows of clinics, &c. held during the session of the Association:

"The clinic operations of the Southern Dental Association, now in session at the Mechanics' Institute, in our city, are exceedingly interesting, even to the general observer.

These operations take place during the morning hour in the convention hall. The operations this morning consisted in one instance of the filling over of an exposed nerve, an operation about the success of which there has not been an entire unanimity of opinion in the profession heretofore.

The operation in question was performed in the most skillful manner by Dr. F. Y. Clarke, of Savannah, Ga., who is an enthusiastic believer in the power of the dentist to save teeth of this kind.

It is contended that in these cases nature, if assisted, will do the work of re-covering the nerve, so as to admit of a perfect filling.

Through the courtesy of Dr. Morrison, of St. Louis, we were shown two newly introduced dental instruments invented by Dr. E. R. E. Carpenter, of Chicago, which are destined to work, and are in truth now working, a great revolution in dentistry. The new instruments, or rather the application of a motive power to the old instruments, are a pneumatic burring engine and plugger, by which cavities are cleaned out and filled by the aid of a tiny air apparatus which is worked by the foot upon a pedal.

The old fashioned dental burring instrument was capable of making about eighteen or twenty revolutions a minute, while the new one will make some eighteen hundred.

The plugging machine moves also in a corresponding manner, doing in five minutes what used to require one hour's time.

Dr. Butler, of Louisville, has upon exhibition an improved dentist's chair, which is capable of being moved in any way that the operator or subject may desire. It is in marked contrast with an old chair sitting near it, some twenty-two years old, used for a long time by Dr. J. S. Clarke and our townsman, Dr. Freidrichs, the well-known dentist."

From the kind reception extended to the numbers of the Association at the Atlanta meeting, we felt assured that our New Orleans colleagues would do all in their power to make the meeting in their city one long to be remembered as a pleasant renuion, and we have not been disappointed.

We deeply regret that circumstances beyond our control should have prevented our being present. The next annual meeting will be held in Charleston S. C. where we again feel assured that true Southern hospitality will be extended to all who may attend.

